# Water-efficient genotypes along with conservation measures significantly reduce the green and blue water footprints in sugarcane (*Saccharum* spp.)

**DOI:** 10.1038/s41598-023-40223-4

**Published:** 2023-08-14

**Authors:** A. S. Tayade, S. Vasantha, S. Anusha, R. Arun Kumar, G. Hemaprabha, P. Geetha, V. Krishnapriya, K. Sammi Reddy, Rajan Bhatt, Manzer H. Siddiqui, Mahipal Singh Kesawat

**Affiliations:** 1 ICAR-Sugarcane Breeding Institute, Coimbatore, 641007 Tamil Nadu India; 2 ICAR-National Institute of Abiotic Stress Management, Baramati, 413115 Pune, India; 3 PAU-Regional Research Station, Kapurthala, 144601 India; 4 Department of Botany and Microbiology, College of Science, King Saud University, Riyadh, 11451 Saudi Arabia; 5 Institute of Molecular Biology and Genetics, School of Biological Sciences, Seoul National University, Seoul, 08826 Republic of Korea

**Keywords:** Plant sciences, Conservation biology

## Abstract

Sugarcane crop is irrigated using surface, overhead, and drip irrigation methods. Increased water use in sugarcane is a major concern around the world, implying the need for water accounting, developing water-efficient hybrids and water-saving agro-techniques for long-term conservation and use of water. “Water Footprint (WF)” is a measure of both direct and indirect water usage accountable for any product and/or process. In praxis, ‘Green Water Footprint’ (GWF) and ‘Blue Water Footprint’ (BWF) are extremely crucial for the restoration of essential ecosystem services (ES), such as sugarcane production. The WF metric was used as a priority tool in our study to evaluate water-efficient sugarcane hybrids, germplasm clones, deficit irrigation scheduling, crop geometry, and water conservation measures. Precise and accurate WF quantification would supplement the decision-making processes for managing available water resources in sugarcane agriculture. In split plot experimental design two research investigations on water management in sugarcane were undertaken at the ICAR-Sugarcane Breeding Institute, Coimbatore, Tamil Nadu, India. The major objective of the research trails was to find out suitable sugarcane hybrids and agronomic management practices to minimise water usage in sugarcane cultivation in water stressed and drought prone areas of tropical India. Our investigation comprised two phases; the first one being assessment of the impact of deficit irrigation scheduling, planting techniques and water conservation measures in sugarcane production, while the second phase dealt with genotypic evaluation under variable irrigation scheduling. Results showed that BWF reduced significantly in the first ratoon crop due to deficit irrigation scheduling coupled with planting of two budded setts and application of sugarcane trash at the rate of 5 t ha^−1^. Sugarcane hybrids viz., Co 85019, Co 10026, Co 12009, Co 13014, Co 14002, Co 14025, Co 15015, and Co 15018 were more water efficient, with a lower total WF. Among the germplasm clones, Fiji 55, ISH 111, ISH 107, Pathri, and Gungera exhibited lower GWF, BWF and total WF.

## Introduction

Water is one of the most valuable natural resources, which is becoming increasingly scarce. There is a mismatch between water usage and availability as the global water demand is increasing at an alarming rate across agriculture, industry, manufacturing, and allied sectors apart from domestic household use. Fresh water availability accounts for approximately 2.5% of the total available water^1–4^, which is retained in rivers, lakes, ponds, and subsurface groundwater. Agriculture, industry, and domestic household use account for 69.0, 23.0, and 13.0% of global fresh water usage, respectively, emphasising the importance of promoting sustainable water use across value chains. Consumption of drinking water and producing an average persons’ daily meal requires 2–4 l, and 2000–5000 L of water, respectively. Human water consumption has increased dramatically over the last three decades, wherein the worldwide water demand, which is now at 4600 km^3^ per year, is predicted to surge by 20.0–30.0% by 2050, reaching 5500–6000 km^3^ per year^5^. Further, global water demand in agriculture would also increase up to 60.0% by 2025^6^, causing a strain on the global available water resources. In India, only 4.0% of the world’s freshwater resources are available, of which 78.0% is consumed for agriculture. India’s population growth, urbanisation, and rapid industrialisation have reduced the per capita water availability from 5888 m^3^ (in 1951) to 1500 m^3^ (by 2025), placing the country in the moderately water-stressed category. In areas classified as moderately or highly water-stressed, the annual total water extraction exceeds 20.0–40.0% of available water, with per capita water availability below 1700 and 500 m^3^ per person per year, respectively^7^. Irrigation management is crucial in water-stressed areas to address long-term resource constraints. In order to manage the world’s freshwater resources judiciously, it is indispensable to quantify the amount of water consumed by every product and/or service. The term “water footprint” (WF) coined by Arjen Y. Hoekstra in 2002 is now widely used as an international metric to evaluate the impact of freshwater resources on global food, non-food, and commercial production, consumption, and trade. The total water footprint (TWF) comprises green, blue and grey water footprints. In fact, WF is an indicator that considers both the direct and indirect water use for a consumer or product^8,9^. The global average WF is approximately 1385 m^3^ per year^10^, emphasising the importance of adopting a balanced approach to the judicious use of water in agriculture, industry, and domestic sectors in order to effectively meet future water demand. In this context, India’s flagship programme *Pradhan Mantri Krishi Sinchayee Yojana (PMKSY)* was launched in 2015 with the goal of providing some form of protective irrigation to every agricultural farm, based on the principle of *Per Drop More Crop*.

India is second only to Brazil, in terms of area under sugarcane cultivation (5.06 Mha), with a total production of 405.30 Mt of cane per year. India’s sugar demand is predicted to reach 48 Mt by 2050^11^, and the strategies for achieving the target comprise water-intensive agricultural practices. Sugarcane is one of the most efficient photosynthesizing crop per se, owing to its C_4_ metabolism that promotes two to three times the growth rate and water use efficiency as compared to C_3_crops^12^. Sugarcane requires between 1500 and 2000 mm of irrigation water per crop cycle to produce a yield of 100–150 t ha^−1^^13^. Water requirement of 12,000 m^3^ per hectare per year is met by scheduling irrigation at regular intervals throughout the sugarcane crop cycle. Sugarcane agriculture in India requires a huge quantity of water (20 ML ha^−1^ year^−1^)^14^, hence priority must be given to efficient irrigation systems/practices that conserve water without jeopardising cane yield. It is apparently paradoxical that, despite having a low field-level application efficiency of about 30.0–50.0%, flood irrigation is extensively used by farmers in approximately 80.0% of the world’s irrigated fields^15,16^. On the other hand, drip irrigation has higher efficiency (70.0–90.0%) due to lower surface runoff and deep percolation losses^17^, and may be the irrigation method of choice in sugarcane. Micro irrigation systems, such as drip and sprinkler irrigation, greatly reduce total irrigation water requirement^18,19^. When compared to conventional irrigation, drip system enhances water use efficiency (WUE) by 60.0–200.0%, saves about 20.0–60.0% water, and decreases fertilizer requirement by 20.0–33.0%, with 7.0–25.0% increment in cane yield^20^. The impact on water saving in sugarcane under tropical Indian conditions is about 31.0 and 23.0% due to subsurface and surface drip irrigation, respectively, as compared to conventional surface irrigation^21^. Micro irrigation systems account for more than 25.0% of total production costs in sugarcane^22,23^, which is the main impediment to its expansion in India. Currently, about 8.7 Mha of agricultural land in India is installed with micro irrigation (45.0% drip and 54.0% sprinkler), accounting for merely about 13.0% of the potential agricultural land area. It is imperative to adopt low-cost, environment friendly irrigation management practises that conserve water while lowering the operational costs and WF in sugarcane. This underpins the significance of designing water-saving and economically viable climate-resilient irrigation management techniques for sugarcane farming. Deficit irrigation scheduling could be a viable option for fulfilling a portion of the crop’s water requirements and allowing it to efficiently absorb moisture from the soil^24,25^. In this context, the combination of water-efficient sugarcane hybrids with deficit irrigation scheduling^26^, in-situ trash mulching and composted coir pith application in furrows at planting time is key to achieve water economy, sustainability, and overall profitability in sugarcane farming. Earlier research revealed that daily water consumption for sugarcane varied between 2 and 6 mm depending on the prevailing climate and phenophase, implying that the soil plant atmospheric continuum has a strong influence on evapotranspiration, biomass accumulation and yield^27^. With this background, two independent field experiments were carried out at ICAR-Sugarcane Breeding Institute, Coimbatore, India, with the following objectives:

1. To measure the GWF and BWF of sugarcane production to determine its impact on water resources in tropical Indian condition
2. To develop agronomic management strategies to minimize GWF and BWF in sugarcane agriculture and to identify water efficient sugarcane hybrids.

## Materials and methods

### Experimental site, soil and weather conditions

Two independent experiments were conducted at ICAR-Sugarcane Breeding Institute, Coimbatore, India (11° N, 77° E at an altitude of 427 m above MSL) for assessing the variation in GWF and BWF due to different planting techniques and water conservation measures (Experiment-I), and to identify water efficient sugarcane hybrids and species clones (Experiment-II)under deficit irrigation scheduling. The typic Haplustalf soil, with low organic carbon, medium available N and available P and high K was chosen for this experiment. The soil had a sandy clay texture with moderate drainage, slightly alkaline in reaction with an EC of 392.8 µS cm^−1^. Soil moisture characteristics of 30.6% field capacity and 9.8% permanent wilting point implied 20.7% of available water content. The experimental site with a mean rainfall of 674.2 mm over 60 years represented a tropical wet and dry climate. During the experimental period, rainfall ranged from 356.0 to 832.4 mm (Table 1), mostly concentrated during the northeast monsoon (Oct–Dec). Average maximum and minimum temperatures of 33.2 and 21.8 °C, respectively, with relative humidity between 60.9 and 84.4% was observed during the experiment. Temperature regime recorded during crop growth was optimal (32–33 °C)^28^, equivalent to sugarcane niches in the tropical and sub-tropical regions of the world, whereas higher temperatures resulted in significant yield reduction^29–31^.

**Table 1:** Irrigation scheduling and Rainfall received during the experimentations.

Crop type/crop season	Irrigation scheduling treatments	No of irrigations given	Quantity of irrigation water applied (mm)	Quantity of irrigation water (Lakh liters)	Rainfall during the crop period (mm)	% of the annual rainfall
Plant crop/2013–2014	I_100_: Full irrigation at recommended intervals with 100% crop ET replacement	30	1470.0	147.0	356.0	51.0
	I_75_: Deficit irrigation scheduling at recommended intervals with 75% crop ET replacement	30	1857.0	185.7		
Ratoon (I)/2014–2015	I_100_: Full irrigation at recommended intervals with 100% crop ET replacement	22	1031.0	103.1	537.0	77.0
	I_75_: Deficit irrigation scheduling at recommended intervals with 75% crop ET replacement	22	1375.0	137.5		
Ratoon (II)/2015–2016	I_100_: Full irrigation at recommended intervals with 100% crop ET replacement	20	783.6	78.4	679.0	98.0
	I_75_: Deficit irrigation scheduling at recommended intervals with 75% crop ET replacement	20	1038.0	103.8		
Plant crop/2019–2020	I_0_: Full irrigation at recommended intervals with 100% crop ET replacement,	31	1289.2	128.9	832.4	123.5
	I_1_: Deficit irrigation scheduling at recommended intervals with 50% crop ET replacement	31	760.7	76.1		
	I_2_: Deficit irrigation scheduling by skipping alternate irrigations with 50% crop ET replacement	22	651.6	65.2		
Plant crop/2020–2021	I_0_R_1_: Full irrigation at recommended intervals with 100% crop ET replacement,	31	1213.3	121.3	624.3	92.6
	I_1_ R_1_: Deficit irrigation scheduling at recommended intervals with 50% crop ET replacement	31	860.7	86.1		
	I_2_ R_1_: Deficit irrigation scheduling by skipping alternate irrigations with 50% crop ET replacement	22	850.8	85.1		

Mean (60 years) rainfall of experimental site: 674.2 mm.

### Experiment I: assessing the impact of planting techniques and water conservation measures under deficit irrigation scheduling on GWF and BWF in sugarcane

**Figure 1:**
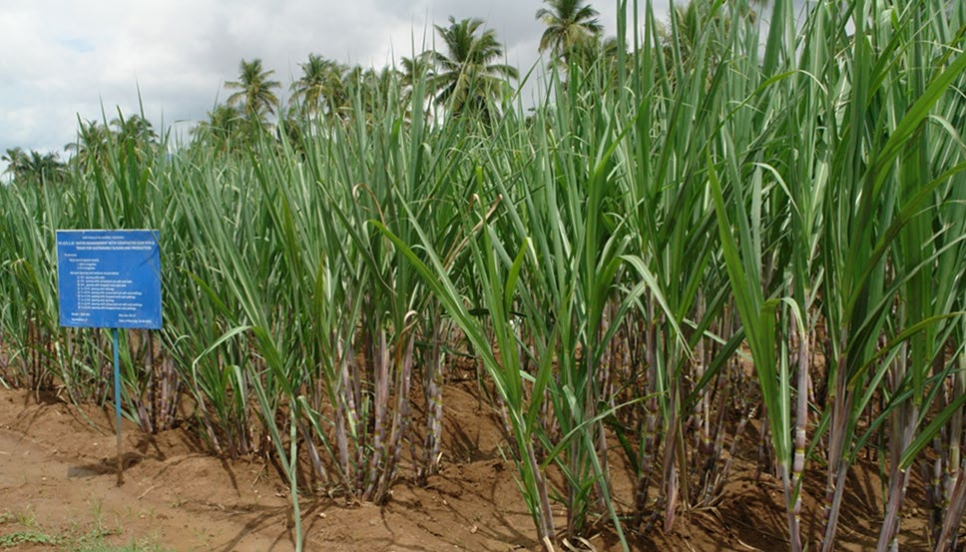
Field view of experiment-I.

The experiment was laid out in a split–split plot design, with irrigation scheduling treatments in main plots, planting techniques in subplots, and conservation measures accommodated by splitting the subplots (Table 2). Three factors including two levels of irrigation scheduling, three types of crop geometry, and three conservation measures, were replicated three times to obtain a total of eighteen (2 × 3 × 3 = 18) treatment combinations (Fig. 1).

**Table 2:** Details of Irrigation scheduling, planting techniques and moisture conservation measures followed in plant-ratoon-ratoon crop cycle (2013–2014, 2014–2015, 2015–2016).

Planting techniques	Irrigation Scheduling treatments and moisture conservation measures					
	Full irrigation at recommended intervals with 100% crop ET replacement			Deficit irrigation scheduling at recommended intervals with 75% crop ET replacement		
150 × 45 cm (BSS)	CCP @ 10 t/ha	Sugarcane trash 5 t/ha	Control	CCP @ 10 t/ha	Sugarcane trash 5 t/ha	Control
150 × 60 cm (BCS)	CCP @ 10 t/ha	Sugarcane trash 5 t/ha	Control	CCP @ 10 t/ha	Sugarcane trash 5 t/ha	Control
90 cm (two budded setts)	CCP @ 10 t/ha	Sugarcane trash 5 t/ha	Control	CCP @ 10 t/ha	Sugarcane trash 5 t/ha	Control

*ET* evapotranspiration, *BCS* bud chip settling, *CCP* composted coir pith.

Dimensions of the main plots were 81.0 × 6.0 m^2^, whereas the sub-plots and sub-sub plots were 27.0 × 6.0 m^2^ and 9.0 × 6.0 m^2^ in size, respectively. Sugarcane was planted in March 2013 and harvested during maturity at 12th month (March 2014), and two ratoon crops were raised from the regrowth of harvested stools in 2015 and 2016. Standard package of practices was followed, including application of nitrogen (N), phosphorus (P), and potassium (K) at the rate of 280.0:62.5:120.0 kg ha^−1^ to plant and 350.0:62.5:120.0 kg ha^−1^ to ratoon crops, respectively. Before planting, 62.5 kg ha^−1^ of P fertilizer was applied as a basal dressing in the plant crop, while N and K were applied in two equal splits at 45 and 90 days after planting (DAP).

#### Deficit irrigation scheduling

Two irrigation scheduling treatments (I_100_ and I_75_) were imposed during the plant-ratoon-ratoon crop cycle. In the I_100_ treatment, irrigation was scheduled at recommended intervals with 100% crop evapotranspiration (ET) replacement, whereas in I_75_ treatment, deficit irrigation was scheduled at recommended intervals with 75% crop ET replacement.

#### Planting techniques

**Figure 2:**
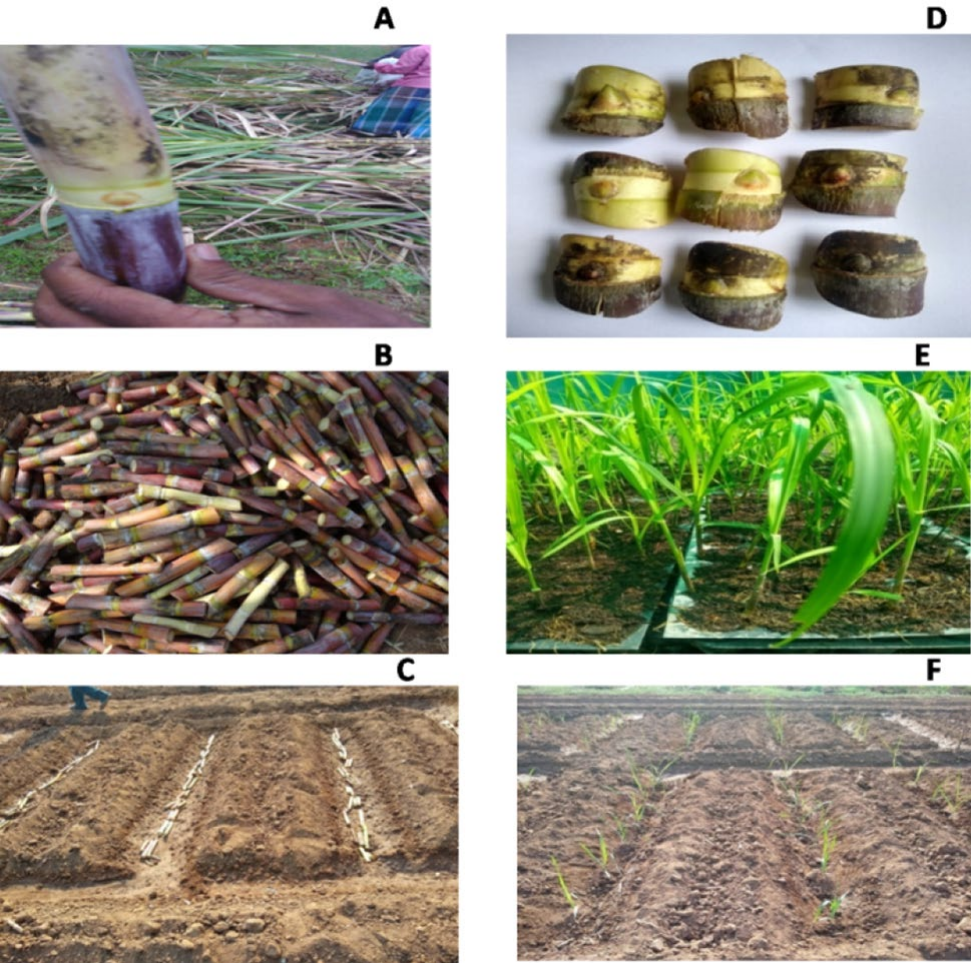
Two budded setts and bud chip settling planting techniques in sugarcane. (**A**) Seed cane with healthy eye bud, (**B**) sizing of two budded setts, (**C**) sett planting at 90 cm row spacing, (**D**) Bud chip scooped from healthy seed cane, (**E**) Bud chip settling raised in pro-trays, (**F**) Bud chip settling planting at 150 × 45 and 150 × 60 cm row spacing.

Two budded setts and bud chip settlings of sugarcane variety Co 86032 were chosen as planting materials, with the following three planting techniques (P_1_, P_2_ and P_3_) (Fig. 2). Popular sugarcane variety Co 86032 developed and released for commercial cultivation by ICAR-SBI in the year 2000, buds for raising settlings were used from cane grown on ICAR-SBI research farm as planting material in our research trials.

In P_1_ (Bud chip settling with crop geometry of 150 × 45 cm) buds were manually scooped from the cane using a bud chip machine, and settlings were raised in the nursery for 30 days before transplanting to the main field. A tractor-drawn ridger was used to prepare the ridges and furrows at 150 cm interval prior to settling transplanting with 18,519 settlings ha^−1^. The field was irrigated immediately after transplanting. In P_2_, (Bud chip settling with crop geometry of 150 cm × 60 cm), 30 days old bud chip settlings at the rate of 13,889 settlings ha^−1^ were transplanted to the main field. In P_3_ (two budded setts with row spacing of 90 cm), 75,000 two-budded setts ha^−1^ was planted in furrows at 90 cm row spacing.

#### Conservation measures

**Figure 3:**
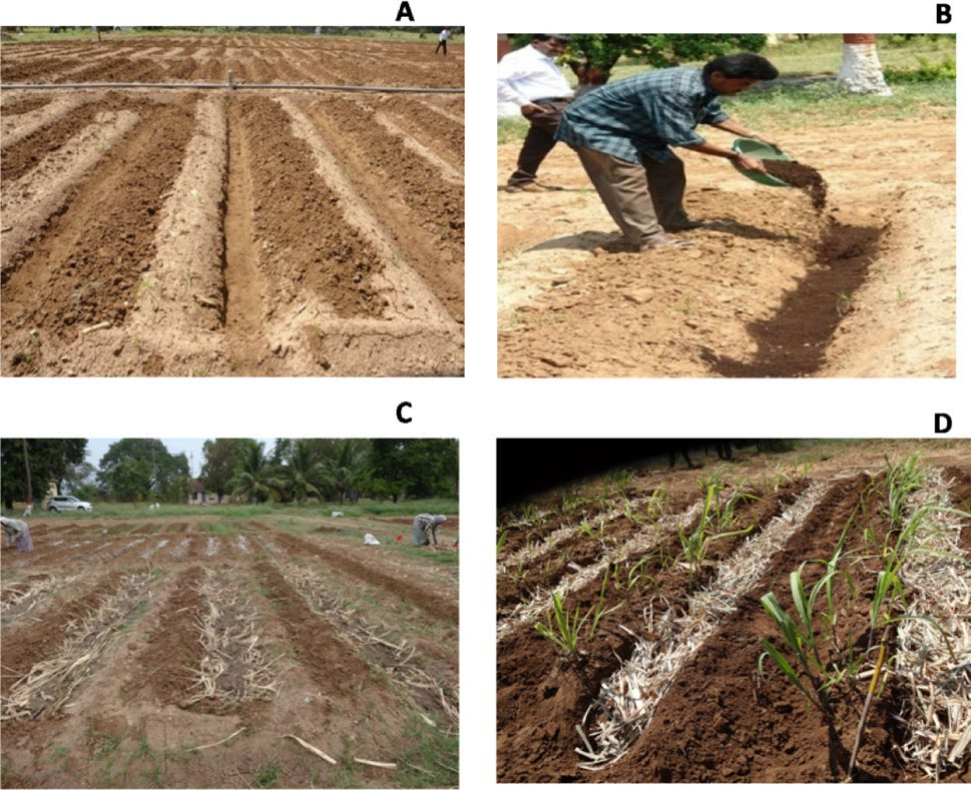
Imposition of water conservation treatments in sugarcane. (**A**) Control plot without water conservation measures, (**B**) app location of composted coir pith, (**C**) application of sugarcane trash in plant crop, (**D**) application of sugarcane trash in ratoon crop.

Figure 3 depicts the imposition of two water conservation measures C_1_: composted coir pith at10 t ha^−1^ and C_2_: sugarcane trash at 5 t ha^−1^ (application in furrow at planting). C_3_ was the control, without any conservation measure.

### Experiment II: assessing the variation among commercial hybrids and species clones of sugarcane under deficit irrigation scheduling for GWF and BWF

**Figure 4:**
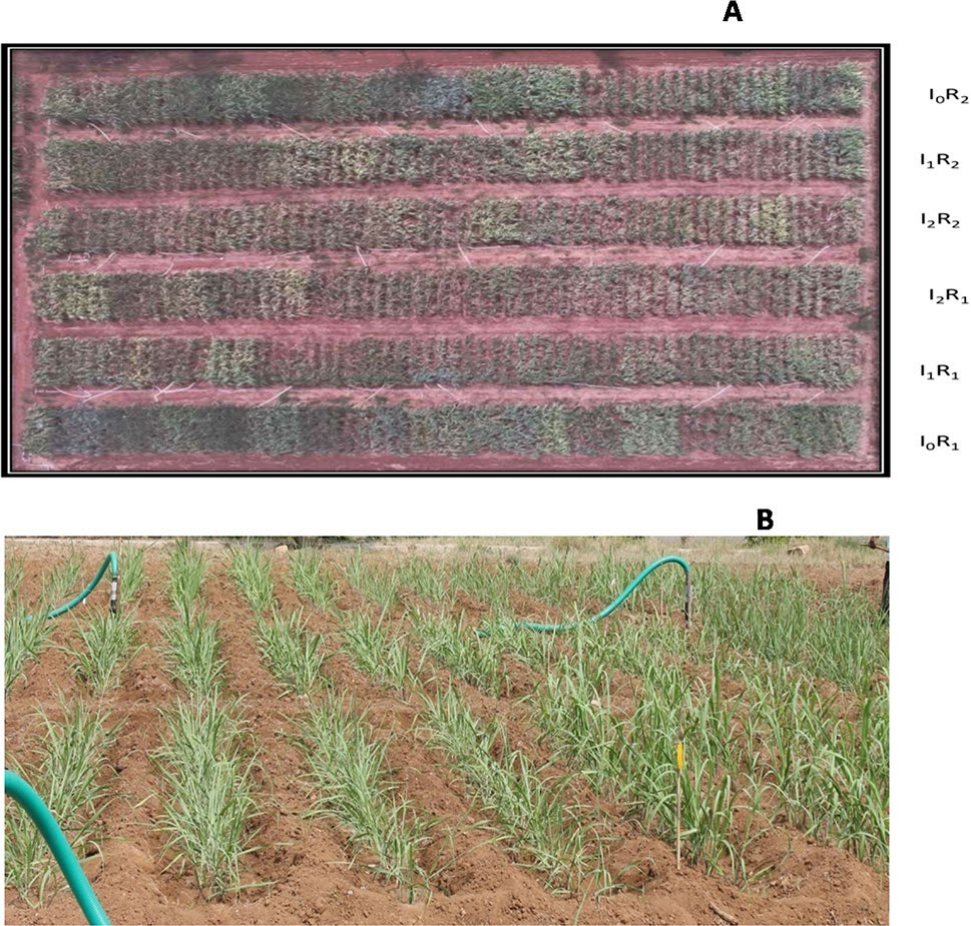
(**A**) Aerial view of field layout of commercial sugarcane hybrids recorded with a drone. I_0_ = full irrigation at recommended intervals with 100% crop ET replacement, I_1_ = irrigation at recommended intervals with 50% crop ET replacement, I_2_ = skipping alternate irrigations with 50% crop ET replacement, R_1_ and R_2_ = replications 1 and 2), field view of commercial sugarcane hybrids in experimental plot (**B**).

**Figure 5:**
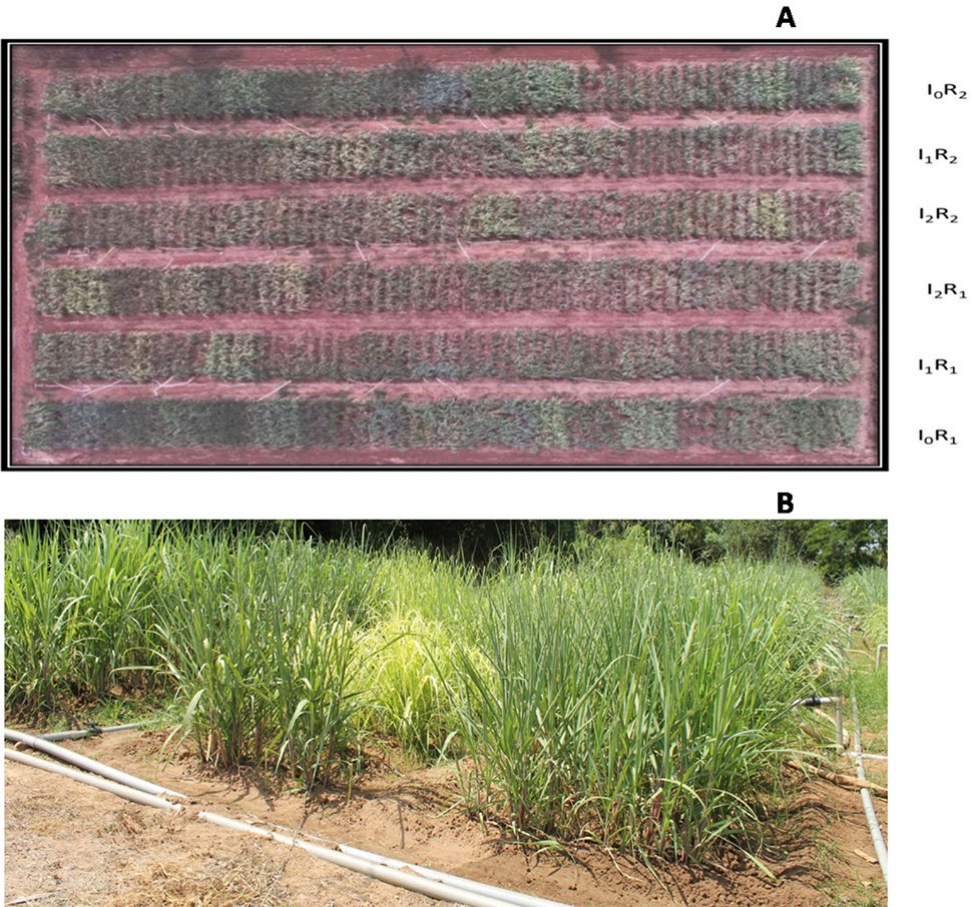
Aerial view of field layout of sugarcane species clones recorded with a drone: I_0_ = full irrigation at recommended intervals with 100% crop ET replacement, I_1_ = irrigation at recommended intervals with 50% crop ET replacement, I_2_ = skipping alternate irrigations with 50% crop ET replacement, and R_1_ and R_2_ = replications 1 and 2 (**A**), field view of sugarcane species clones in experimental plot (**B**).

Experiment-II was conducted independently for commercial hybrids and species clones during the crop seasons 2019–2020 and 2020–2021 in split plot design with two replications wherein deficit irrigation scheduling was the main plot, and sugarcane genotypes were accommodated as subplots (Figs. 4 and 5).

A tractor-drawn ridger was used to open 25 cm deep furrows at 90 cm row spacing, wherein the two budded setts were planted at the rate of 75,000 ha^−1^ and covered with light soil. The main plots measured 81.0 × 6.0 m^2^ in size, whereas the sub-plots were 27.0 × 6.0 m^2^. A recommended fertilizer dose of 280:62.5:120 kg NPK ha^−1^ was applied to the sugarcane crop, with 62.5 kg ha^−1^ of P applied as a basal dressing before planting, while N and K were applied in two equal splits 45 and 90 DAP. Mature sugarcane stalks were harvested manually at 12th month.

#### Irrigation scheduling

The effect of deficit irrigation scheduling on sugarcane hybrids and species clones was investigated by following three irrigation schedules such as; I_1_: Full irrigation at recommended intervals with 100% crop ET replacement, I_2_: Deficit irrigation scheduling at recommended intervals with 50% crop ET replacement and I_3_: Deficit irrigation scheduling by skipping alternate irrigations with 50% crop ET replacement.

#### Commercial sugarcane hybrids and species clones

Commercial sugarcane hybrids and species clones as listed in Table 3 were evaluated for their response to varied irrigation schedules. Popular sugarcane variety Co 86032 developed and released for commercial cultivation by ICAR-SBI in the year 2000, was used as planting material in our research trials. After the first world collection of sugarcane germplasm maintained at the USDA’s Sub-tropical Horticulture Research Station, Miami, Florida with nearly 1100 accessions, the second world collection was established in India during 1956 by ICAR-SBI at its Research Centre, Kannur (Kerala). Today, this is the largest and the most diverse collection of *Saccharum* germplasm (3360 accessions) in the world. In addition to the site at Kannur, the collections of *Erianthus fulvus* and *Miscanthus nepalensis* from high altitude regions (north eastern Himalaya) are maintained at ICAR-IARI Regional Research Station, Wellington, Nilgiris (Tamil Nadu) and ICAR-SBI, Research Centre, Agali (Kerala). Another collection of Indian *S. spontaneum* and *Erianthus* species and a working germplasm collection (2300 accessions) are also maintained at ICAR-SBI, Coimbatore. Realizing the importance of wide hybridization in sugarcane improvement programmes, the Institute established a National Distant Hybridization Garden facility at its Research Centre at Agali (Kerala) in the year 2000 where about 1380 clones are maintained for utilization by breeders from different research stations in India^51^. Material for the *Saccharum* species clones used in this trial were officially transferred from ICAR-SBI Research Centre, Kannur, in compliance with relevant institutional, national, and international guidelines and legislations.

**Table 3:** Details of commercial hybrids, species clones of sugarcane screened against the deficit irrigation scheduling done during 2018–2019 and 2020–2021 crop season.

Irrigation scheduling treatments	2019–2020		2020–2021	
	Commercial hybrids (18 nos)	Species clones (16)	Commercial hybrids (19)	Species clones (19)
Full irrigation at recommended intervals with 100% crop ET replacement	Co 15007, Co 15021, Co 15015, Co 14025, Co 14002, Co 8021, Co 15018, Co 16018, Co 85019, Co 11015, Co 12009, Co 95020, Co 13014, CoM 0265, Co 10026, Co 86032, Co 09004, Co 0212	ISH107, Kheli, Nargori, ISH 111, Khakkai, ISH 23, ISH 58, Lalri, ISH 9, Matna shaj, IK 76100, Dhaurgalig, Pathri, Fiji 55, 57 NG 77, Gungera	Co 09004, Co 10026, Co 11015, Co 12009, Co 13014, Co 14002, Co 14025, Co 15007, Co 15015, Co 15018, Co 15021, Co 16018, Co 85019, Co 94008, CoM 0265, Co 0212, Co 86032, Co 62175, Co 99004	Lalri, ISH9, ISH58, ISH107, ISH111, Kheli, Nargori, ISH23, Khakkai, MatnaShaj, Dhaurgalig, Pathri, Gungera, Reha, Fiji 55, Twc-50, Twc-82, Twc-13, Cym 08-922
Deficit irrigation scheduling at recommended intervals with 50% crop ET replacement				
Deficit irrigation scheduling by skipping alternate irrigations with 50% crop ET replacement				

### Measurement of irrigation water and irrigation scheduling

**Figure 6:**
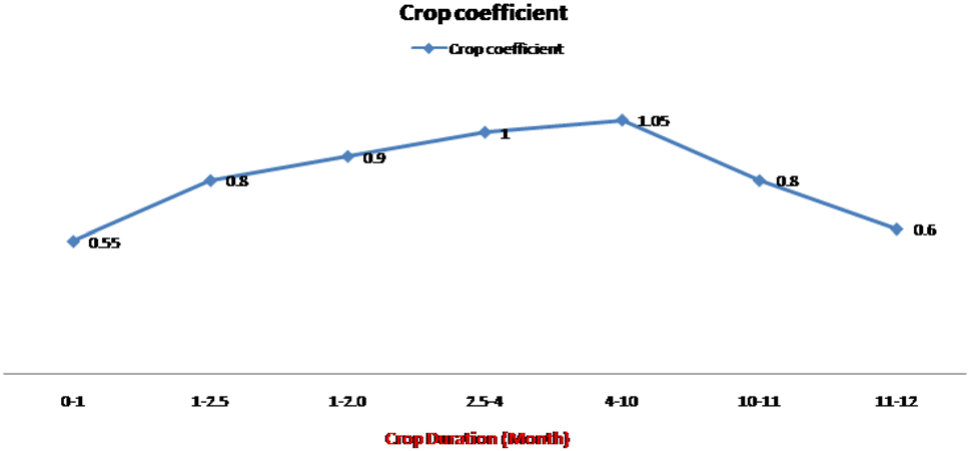
Crop coefficients used for scheduling irrigation during different developmental stages of sugarcane.

In both the experiments I and II, irrigation was scheduled using the standard protocol, which involved measuring evaporation rates with a ‘class A’ pan evaporimeter and applying irrigation water based on cumulative pan evaporation (CPE). Equation (1) was used to calculate the amount of irrigation water required for each treatment plot (Q).

1 $$\mathrm{Q }=\mathrm{CPE }\times {\mathrm{K}}_{\mathrm{c}} \times \mathrm{A}$$

where Q is the amount of irrigation water required for area A, CPE and K_c_ are cumulative pan evaporation and crop coefficient as per crop stage, respectively. The crop coefficient factors used for scheduling irrigation to sugarcane crop are given in Fig. 6.

**Figure 7:**
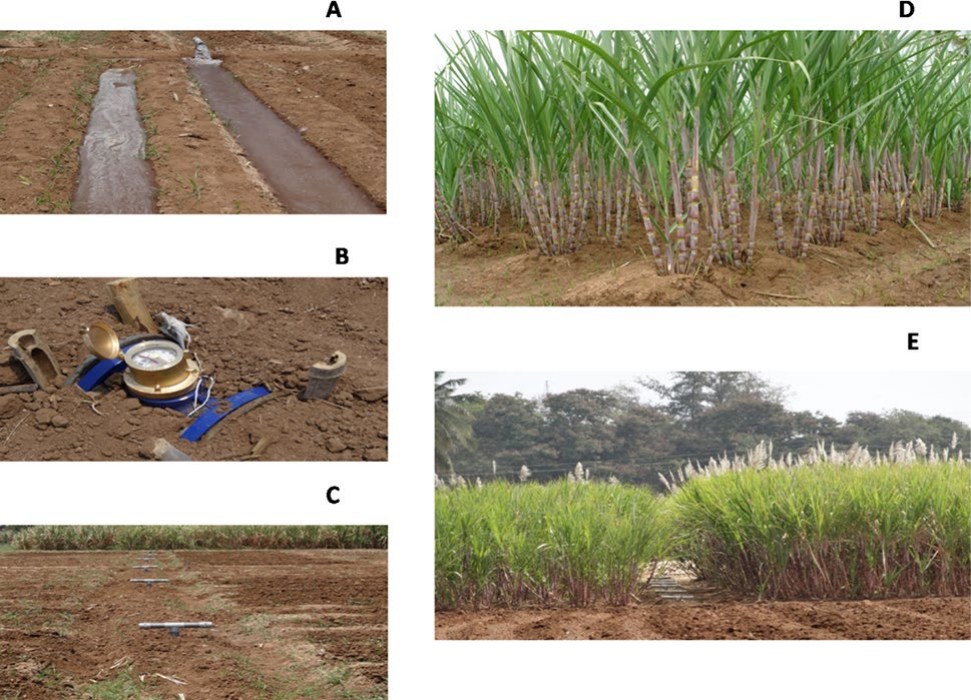
(**A**) Irrigation scheduling with furrow irrigation system, (**B**) irrigation water measurement with water meter, (**C**) conveyance of irrigation water through PVC pipeline in main field, (**D**) field view of furrow irrigated crop deficit irrigation scheduling (I_75_) at recommended intervals with 75% crop ET replacement, (**E**) deficit irrigation scheduling (I_100_) at recommended intervals with 100% crop ET replacement.

A measured quantum of water was applied according to the treatments, which was monitored regularly using a water meter (Fig. 7).

Sugarcane crop water requirement vary depending on crop stage; hence irrigation scheduling was done according to the schedule given in Table 4 during the experiment. The first two irrigations on 1 and 3 DAP were applied with a uniform amount of irrigation water. From the third irrigation onwards, deficit irrigation scheduling (I_100_ and I_75_, i.e. 100 and 75% CPE) was initiated in experiment-I. In experiment-II, conservation measures were not a part of the treatment, hence, deficit irrigation was initiated at 60 DAP to ensure uniform crop establishment. The quantity of irrigation water applied through furrow in each treatment is given in Table 1. Water is applied by running small streams between crop rows, thereby water infiltrates the soil and distributes laterally, irrigating the spaces between the furrows.

**Table 4:** Irrigation scheduling in sugarcane according to different phenophases.

S. no	Duration in days	Sugarcane phenophases	Irrigation scheduling interval (days)
1	0–35	Germination	7
2	36–100	Tillering	10
3	101–270	Grand growth	7
4	271–365	Maturity	15

### Measurement of green, blue and total water footprints

WF for any product or process includes three components: blue, green, and grey; however, for experiments I and II, only green, blue, and total water footprints were measured. At harvest, cane yield was recorded independently for each treatment to calculate the green, blue, and total water footprints.

#### Measurement of GWF

GWF refers to ratio of loss of green water resources (profile stored soil moisture or rainwater in so far as it does not become runoff) due to evaporation or evapotranspiration during the crop growth period to the quantity of economic crop yield produced (Eq. 2). In simpler terms, GWF refers to the volume of rainwater consumed for cane yield production. While calculating GWF, only effective rainfall received by the sugarcane crop was considered.

2 $$\mathrm{Green\,\, water \,\,footprint }\,\,({\mathrm{m}}^{3}{\mathrm{t}}^{-1}) =\frac{\mathrm{Volume\,\, of \,\,green \,\,water \,\,use }\,\,({\mathrm{m}}^{3}{\mathrm{ha}}^{-1})}{\mathrm{Cane \,\,yield }\,\,(\mathrm{t }{\mathrm{ha}}^{-1})}$$

#### Measurement of BWF

BWF is the volume of blue water (surface and ground water) consumed during the life cycle of a crop to the quantity of economic crop yield produced (Eq. 3). In simpler terms, BWF refers to the volume of surface or groundwater consumed for the cane yield production. While calculating BWF, amount of irrigation water applied to the sugarcane crop was considered.

3 $$\mathrm{Blue\,\, water\,\, footprint }\,\,({\mathrm{m}}^{3}{\mathrm{t}}^{-1}) =\frac{\mathrm{Volume\,\, of \,\,blue \,\,water \,\,use }\,\,({\mathrm{m}}^{3}{\mathrm{ha}}^{-1})}{\mathrm{Cane\,\, yield }\,\,(\mathrm{t }{\mathrm{ha}}^{-1})}$$

#### Measurement of total water footprints (TWF)

TWF is the ratio of the volume of green (rainwater) plus blue water (surface and ground water) consumed during the life cycle of a crop to the quantity of economic crop yield produced (Eq. 4).

4 $$\mathrm{Total\,\, water \,\,footprint }\,\,({\mathrm{m}}^{3}{\mathrm{t}}^{-1}) =\frac{\mathrm{Volume \,\,of \,\,green \,\,water \,\,use }\,\,({\mathrm{m}}^{3}{\mathrm{ha}}^{-1})+\mathrm{Volume \,\,of \,\,blue \,\,water \,\,use }\,\,({\mathrm{m}}^{3}{\mathrm{ha}}^{-1})}{\mathrm{Cane\,\,Yield }( \,\,(\mathrm{t }{\mathrm{ha}}^{-1})}$$

### Statistical analysis

For experiments I and II, analysis of variance was performed for GWF, BWF and TWF using a split-split design and a split plot design, respectively^32^. Least significant differences (LSD) at P < 0.05 were used for comparison of means.

## Results

### Variation among commercial hybrids and species clone for sugarcane WF

**Figure 8:**
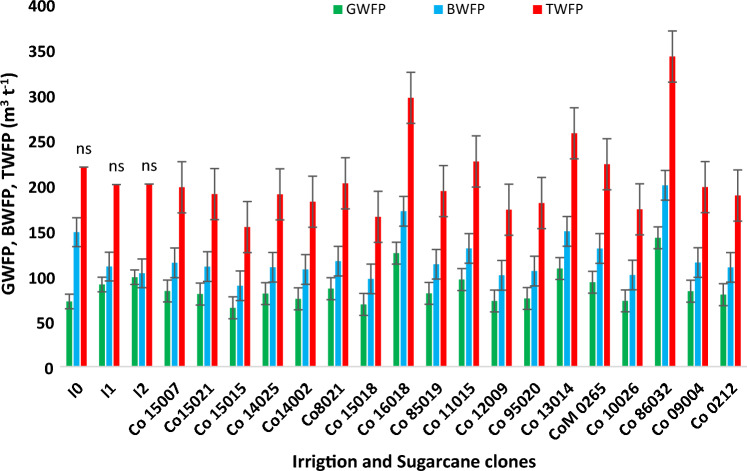
Impact of deficit irrigation scheduling, and Variation among sugarcane commercial hybrids for green, blue and total water footprint during the cropping season 2019–2020.

**Figure 9:**
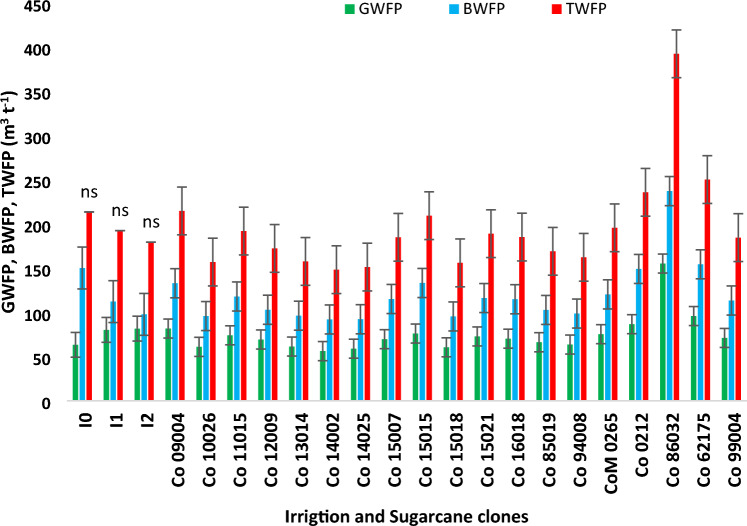
Variation among sugarcane commercial hybrids for green, blue and total water footprint during the cropping season 2020–2021.

**Figure 10:**
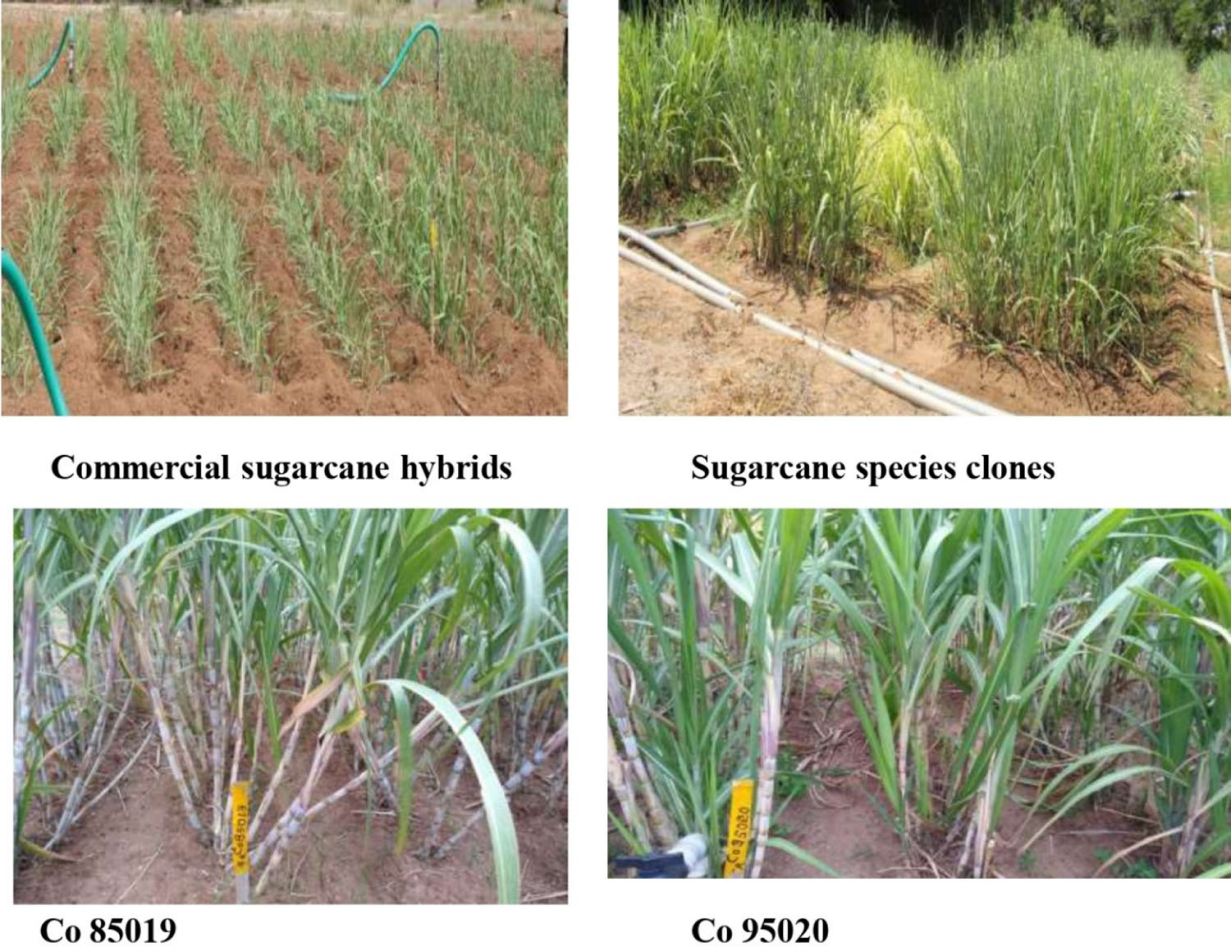
Promising commercial hybrids and species clones exhibiting lower water footprints.

Commercial sugarcane hybrids and germplasm clones recorded a significant variation for GWF (Figs. 8, 9 and 10). During the cropping season 2019–2020 with a total of 832.4 mm precipitation over 49 rainy days, the hybrids responded differently in terms of GWF due to their significantly varying cane yield. Commercial hybrids including Co 15015, Co 15018, Co 12009, Co 10026 and Co 14002 registered GWF below 75.00 m^3^ t^−1^, which was significantly lower than Co 86032 (142.02 m^3^ t^−1^) and Co 16018 (125.06 m^3^ t^−1^). Likewise, during the cropping season 2020–2021, amount of rainfall received (624.3 mm) was 7.4% less than the 60-year average. Promising commercial hybrids such as Co 14002, Co 14025, Co 15018, Co 10026, Co 13014, Co 85019, and Co 12009 consumed considerably less rain water with GWF lower than 70.00 m^3^ t^−1^. BWF accounted for a greater proportion of the TWF as evidenced in Figs. 8 and 9. During the 2019–2020 cropping season, commercial hybrids Co 15015, Co 15018, Co 12009, Co 10026 and Co 14002 recorded significantly lower BWF (< 110 m^3^ t^−1^), in comparison to hybrids consuming greater amount of blue water such as Co 86032 (199.90 m^3^ t^−1^) and Co 16,018 (171.29 m^3^ t^−1^). Similarly during the cropping season 2020–21, the commercial hybrids Co 14002, Co 14025, Co 15018, Co 10026, Co 13014, and Co 85019 exhibited BWF lower than 100 m^3^ t^−1^. TWF exhibited a similar trend wherein the commercial hybrids such as Co 15015, Co 15018, Co 12009, Co 10026, and Co 14002 recorded values lower than Co 86032341.93 and 392.42 m^3^ t^−1^).

**Figure 11:**
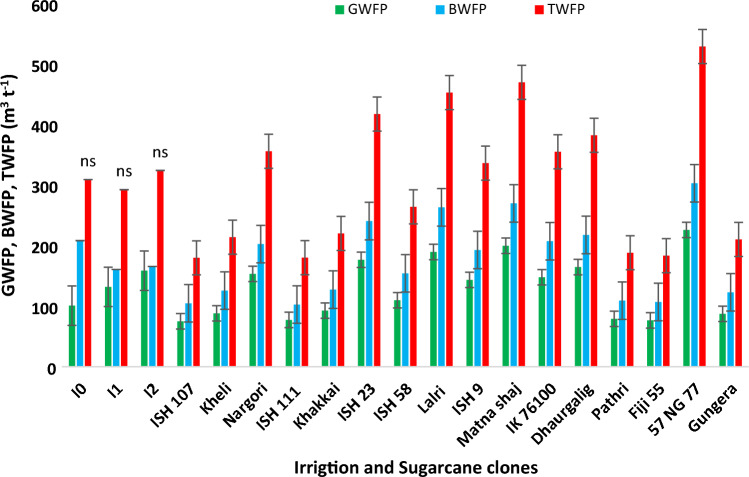
Variation among germplasm clones for green, blue and total water footprint during the cropping season 2019–2020.

**Figure 12:**
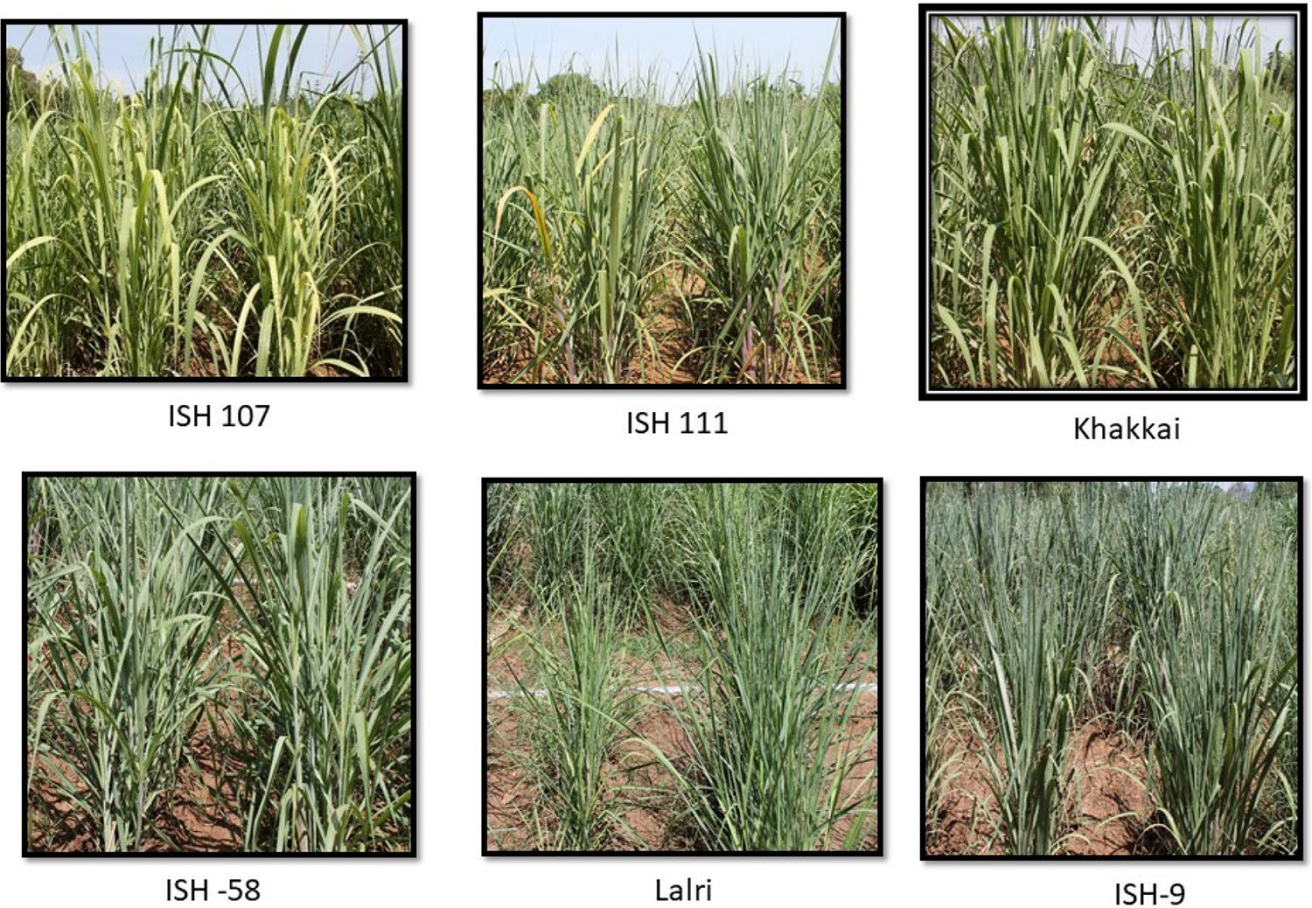
Promising sugarcane species clones exhibiting lower water footprints.

**Figure 13:**
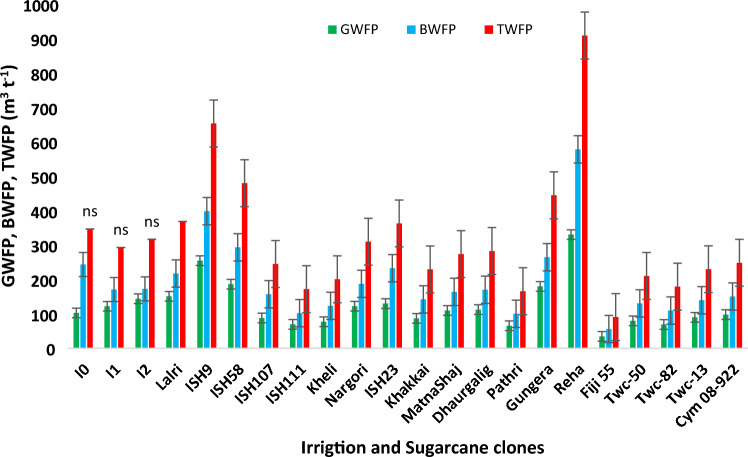
Variation among germplasm clones for green, blue and total water footprint during the cropping season 2020–2021.

Germplasm clones Fiji 55, ISH 111, ISH 107, Pathri and Gungera recorded lower GWF, BWF and TWF during both the cropping seasons (Figs. 11, 12, 13). Our investigation on the varietal variation in WF resulted in identification of potential genotypes that could be valuable sources for dissecting the phenotypic traits and molecular mechanisms of higher water productivity in sugarcane.

### Effect of deficit irrigation scheduling on sugarcane WF

Efficient irrigation technologies as well as irrigation scheduling can be optimized in sugarcane for the rational use of limited water. Results from experiment-I revealed that deficit irrigation (I_75_) had no significant impact on the GWF in both plant and ratoon crops (Table 5), while a moderate reduction was observed in BWF and TWF in the plant crop (2013–2014)with a substantial reduction in ratoon crop growth during 2014–2015 and 2015–2016. Deficit irrigation (I_75_) reduced yield proportionate to the magnitude of water stress, along side saving significant quantum of irrigation water 387, 344 and 255 mm in plant (387 mm), ratoon I (344 mm), and ratoon II (255 mm) crops.

**Table 5:** Impact of deficit irrigation scheduling, crop geometry, planting techniques and moisture conservation measures on green, blue and total water footprint (m^3^ t^−1^) in plant-ratoon-ratoon crop cycle.

Treatments	Plant crop			Ratoon crop (I)			Raton crop (II)		
	GWFP	BWFP	TWFP)	GWFP	BWFP	TWFP	GWFP	BWFP	TWFP
Irrigation scheduling (I)									
I_0_	38, 38	205.92	244, 30	56.85	163.70	220.54	74.20	180.70	254.90
I_1_	39.69	168.74	208.44	51.32	110.81	162.13	73.18	133, 63	206.81
t-test (p = 0.005)	NS	NS	NS	NS	27.55	34, 59	NS	19.37	24.41
Planting techniques (P)									
150 × 45 cm	39.73	189.94	229.67	54.21	137.19	191.39	75.59	161.29	236.88
150 × 60 cm	41.91	201, 08	242.99	61.02	155.33	216.35	82.56	175.85	258.40
90 cm	35.47	170.97	206.44	47.02	119.24	166.56	62.91	134.38	197.29
t-test (p = 0.005)	3.99	20.50	24.47	2.85	7.40	10.24	7.57	16.61	24.13
Interaction effect (I × P)									
t-test (p = 0.005)	NS	NS	NS	NS	10.47	14.48	NS	NS	NS
Water conservation measures (C)									
Composted coir pith	31.18	169.55	204.88	49.50	124.97	174, 47	68.71	146.52	215, 22
Sugarcane trash	34.68	188.33	227.47	51.00	129.14	180.13	71.40	152.17	223.57
Control	38.71	204.12	246.76	61.75	157.64	219.40	80.96	172.82	253.78
t-test (p = 0.005)	2.32	10.97	13.27	2.36	6.40	8.75	4.70	10.26	14, 92
Interactions effects (I × C)									
t-test (p = 0.005)	NS	NS	NS	3.34	9.05	12.37	NS	NS	NS
Interactions effects (P × C)									
t-test (p = 0.005)	NS	NS	NS	4.09	11.09	15.51	8.13	17.77	25.84
Interactions effects (I × P × C)									
t-test (p = 0.005)	NS	NS	NS	NS	15.68	21.43	NS	NS	NS

*I*_*0*_ full irrigation at recommended intervals with 100% crop ET replacement, *I*_*1*_ deficit irrigation scheduling at recommended intervals with 75% crop ET replacement, *GWFP* green water footprint, *BWFP* blue water footprint, *TWFP* total water footprint.

**Figure 14:**
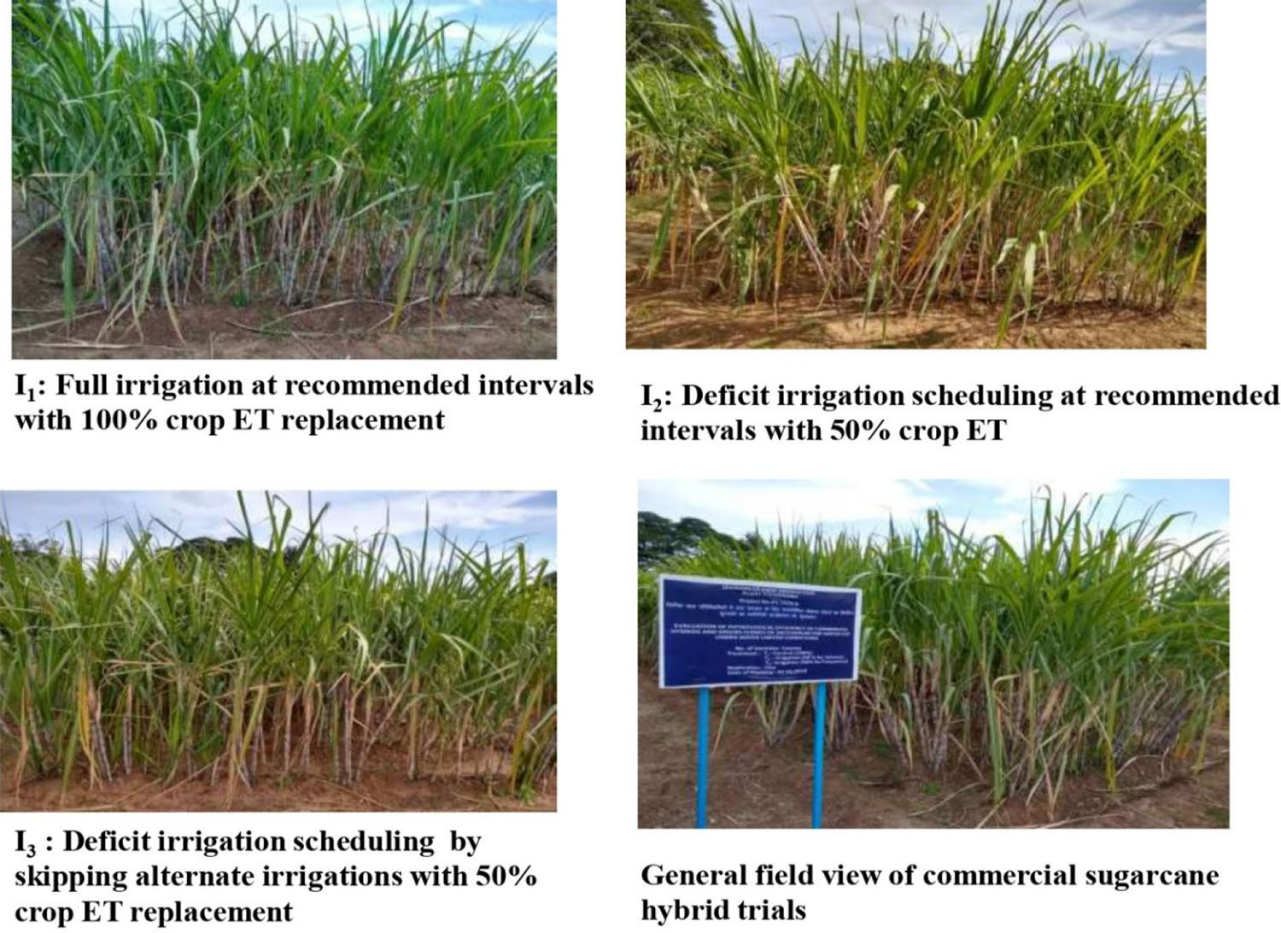
Effects of deficit irrigation scheduling on commercial sugarcane hybrid.

The magnitude of deficit irrigation scheduling was intensified by limiting irrigation water by 50% of crop ET replacement as against full irrigation (100% of crop ET replacement, I_100_), the former being imposed either by reduction in irrigation volume (I_50volume_) or by skipping alternate irrigations (I_50frequency_). Compared to the deficit irrigation scheduling treatments (I_50volume_ and I_50frequency_), I_100_ had a significantly smaller GWF in both cropping seasons, which may be attributed to adequate available soil moisture (ASM) for sugarcane development when irrigation is given at recommended intervals with 100% crop ET replenishment. In contrast, a 50% deficit irrigation schedule may not supply sufficient soil moisture for sugarcane growth, resulting in lower cane yield (Fig. 14), thus recording a higher WF for every ton of harvested cane.

### Effect of planting techniques on sugarcane WF

Our investigations revealed that different planting techniques had a significant impact on the WF of sugarcane (Table 5). Planting of two-budded sugarcane setts significantly reduced the GWF, BWF and TWF in plant crop, ratoon II as compared to bud chip settling transplanting at different crop geometries of 150 × 45 cm and 150 × 60 cm. Among the two test crop geometries, planting of bud chip settling at 150 × 45 cm recorded comparatively lower GWF, BWF and TWF than 150 × 60 cm.

### Effect of water conservation measures on sugarcane WF

Water conservation measures such as CCP and trash incorporation significantly reduced GWF, BWF and TWF in plant and ratoon crops of sugarcane. Reduced water loss and an overall improvement in the micro-climate of the sugarcane ecosystem maybe attributed to the reduction in WF in treatments incorporating water conservation measures.

### Interactive effects of genotypes, deficit irrigation scheduling, planting techniques and water conservation measures on sugarcane WF

#### Interactive effect of deficit irrigation scheduling and commercial hybrids

During the 2019–2020 cropping season, the interactive effect of deficit irrigation scheduling and commercial hybrids was significant, wherein the hybrid Co 12009 responded better to I_50frequency_, with the lowest GWF (66.61 m^3^ t^−1^), BWF (81.17 m^3^ t^−1^) and TWF (147.78 m^3^ t^−1^). Likewise, during the 2020–2021 cropping season, Co 12009 efficiently utilized the rain water and recorded the lowest GWF (39.35 m^3^ t^−1^) at I_100_. In the deficit irrigation scheduling treatment I_50volume_, the commercial hybrids Co 14002 (65.34 m^3^ t^−1^) and Co 14025 (67.30 m^3^ t^−1^) recorded the lowest BWF and TWF.

#### Interactive effect of deficit irrigation scheduling and germplasm clones

During the cropping season 2019–2020, clone ISH 107 at deficit irrigation scheduling I_50frequency_ recorded the lowest TWF (170.12 m^3^ t^−1^), while ISH 111 had the lowest TWF (107.52 m^3^ t^−1^)in the treatment I_50volume_.

#### Interactive effect of deficit irrigation scheduling, planting techniques, and water conservation measures

In ratoon I, significant interactive effects were observed, wherein deficit irrigation scheduling (I_75_) coupled with application of CCP at the rate of 10 t ha^−1^ recorded the lowest GWF (48.73 m^3^ t^−1^), followed by treatment combination of deficit irrigation scheduling (I_75_) and incorporation of sugarcane trash at the rate of 5 t ha^−1^. Likewise, the treatment combination of deficit irrigation scheduling (I_75_), two budded setts planting, and application of sugarcane trash at the rate of 5 t ha^−1^ recorded the lowest BWF (93.88 m^3^ t^−1^).

## Discussion

### Green and blue WFs contributes to TWF of sugarcane under tropical condition

Results of the present investigation revealed that under tropical Indian condition, the share of the BWF to the TWF was significantly higher than GWF. This may be associated with the usage of more irrigation water rather than rain water for sugarcane farming. Sugarcane production necessitates a considerable amount of water (an average of 20 ML ha^−1^) with 80% of the requirement being met from groundwater extraction. According to India’s Central Ground Water Board, about 162 billion cubic metres (BCM) of groundwater per year is accessible for future irrigation, with about 40 BCM per year for sugarcane cultivation, as against the annual demand of 100 BCM^14^. Genotypes with low BWF are essential to combat the ever-increasing constraint of water scarcity in sugarcane agriculture. Our results revealed that commercial hybrids such as Co 14002, Co 14025, Co 15018, Co 10026, Co 13014, and Co 85019 used significantly less water, recording a significantly lower TWF than the global average WF (210.00 m^3^ t^−1^). These hybrids have the potential to alleviate the stresses related to water scarcity in tropical Indian sugarcane cultivation.

The average TWF for sugarcane production comprising green, blue and grey water footprint was 139.00, 57.00 and 13.00 (m^3^ t^−1^), respectively^33^. Reduced WF observed in water efficient sugarcane hybrids may be attributed to an efficient C_4_ photosynthetic process promoting vigorous growth and water^12^. Furthermore, the early production of tillers may be linked to differential response of genotypes to water consumption in terms of GWF and BWF. The yield of a cultivar is mostly governed by its capacity to put forth productive tillers with efficient rate of growth^34^. Tillering is an important yield attributing factor in sugarcane, which acts as storage sink for sucrose accumulation. Sugarcane yield is strongly influenced by the number of millable canes (NMC) per unit area^35^, which is largely determined by tiller production and survival^36^. Earlier research demonstrated that tiller mortality is proportional to NMC^37^. Cultivars exhibiting early and profuse tillering contribute significantly to cane production, whereas cultivars with late-formed tillers usually perish or remain immature, lowering the quality of cane juice. Early tiller production may be linked to the differential response of genotypes to water consumption as observed from GWF and BWF in the present study.

Germplasm clones Fiji 55, ISH 111, ISH 107, Pathri and Gungera recorded lower GWF, BWF and TWF during both the cropping seasons. Internal water deficit occurs when transpiration exceeds absorption, however, the efficient genotypes identified from the present study might have responded better to deficit irrigation scheduling by regulating their stomatal aperture soon after the onset of soil moisture deficit, preventing damage prior to significant changes to leaf water potential, in turn, conserving soil moisture to sustain crop growth. Such desirable traits may be introgressed to commercial sugarcane hybrids through conventional breeding or molecular approaches, in order to obtain high-yielding varieties with low TWF.

### Deficit irrigation scheduling reduces sugarcane WF

Sugarcane growth and development is optimal when soil moisture levels are close to field capacity. Irrigation at 50% depletion of ASM during the vegetative phase (up to 170 DAP) and 75% depletion of ASM during the maturity phase (270–360 DAP) was found to be suitable for sugarcane growth and development^38^.

As a consequence of climate change, drought or insufficient ASM will have a significant impact on sugarcane, as the latter requires a huge quantum of water for optimal growth and development^39^. However, deficit irrigation scheduling (I_50volume_ and I_50frequency_) was effective in considerably reducing BWF and TWF, as compared to full irrigation (I_100_). Several studies have reported that moisture depletion had a negative impact on sugarcane growth and yield, emphasizing the importance of deficit irrigation scheduling for economic crop production, with differential responses to varying soil fertility, climate, genotype and growth conditions^40–42^.

### Crop geometry significantly influenced sugarcane WF

Crop geometry influenced NMC, productivity, and WF, which was evident from our results assessing different planting techniques. This was in agreement to previous reports wherein planting sugarcane with 90 cm row spacing improved cane yield as compared to 150 cm row spacing^43^, owing to significantly higher NMC observed in the former. Increasing row spacing from 90 to 120 cm did not cause much difference in cane yield, whereas further increment of row spacing considerably reduced yield^44^.

### Water conservation measures significantly reduce sugarcane WF

Trash mulching is an effective strategy to maintain soil moisture and minimize the effects of stress in sugarcane. Mulching helped to maintain soil moisture by reducing evaporation from the surface, as well as to moderate the soil temperature, promote germination, decrease weed growth and increase tiller survival. In situ trash mulching helped in conserving 0.70–5.92% of soil moisture, alongside buffering the soil temperature in the top 5 cm soil layers at 25.1–27.2 °C, whereas the daily temperature fluctuation in the control plot (without conservation measures) was significantly higher (26.9–34.0 °C). In situ trash mulching combined with the use of microbial consortia resulted in significantly higher single cane weight, cane height and girth, as well as NMC and cane yield^45^. Incorporation of shredded trash with microbial consortia in sugarcane ratoon crop reduced soil compaction in the rhizosphere, lowered bulk density and soil penetration resistance^46^. It also increased organic carbon, and available nutrients, which in turn resulted in a higher cane yield. In our study, the application of CCP at the rate of 10 t ha^−1^ during planting reduced GWF, BWF and TWF, in agreement with previous reports of CCP along with deficit irrigation schedule increasing the water use efficiency as compared to control^47^. The effectiveness of trash mulching in conserving irrigation water and boosting cane yield culminated in a lower BWF. Our findings corroborate with earlier work wherein 5.6% higher cane yield was reported under sub-surface drip irrigation coupled with trash mulching as compared to conventional surface irrigation without mulching^48^.

### Genotypic variation among commercial hybrids and germplasm clones to WF

Salient findings of our study indicated that Co 12009, Co 14002, and Co 14025 are suitable for deficit irrigation scheduling (I_50volume_), and may be recommended for cultivation in drought-prone areas of tropical India. Germplasm clones such as ISH 107 and ISH 111 identified from our study may be used to develop high yielding sugarcane varieties with low WF. Such efficient genotypes may be climate resilient with the ability to tolerate moderate amounts of stress through morphological, physiological, and biochemical adaptations. Inward rolling of the upper canopy evident in water use efficient sugarcane genotypes resisted the irradiance load, allowing for lesser direct sunlight absorption. Likewise, waxy layer on the leaf surface helps limit water loss from the leaf and nodal areas of the cane. Presence of thick cuticles and waxy leaf surfaces reflect the incident light, thereby reducing the impact of stress^49^. Drought tolerance was associated with less transpiring leaves with a low density of sunken stomata and wide vascular bundles, exhibiting a wide variation among sugarcane cultivars^50^.

## Conclusion

Planting of two budded setts coupled with application of CCP at a rate of 10 t ha^−1^ under deficit irrigation scheduling (I_75_) significantly reduced the BWF and TWF of sugarcane under tropical Indian conditions. Furthermore, deficit irrigation scheduling (I_50_) paired with a water-efficient commercial hybrids and germplasm clones considerably reduced the BWF and TWF, outperforming plant growth under full irrigation (I_100_). The study will be very useful in drought prone areas of tropical conditions and could sustained sugarcane production under water limited conditions. Commercial sugarcane hybrids such as Co 85019, Co 10026, Co 12009, Co 13014, Co 14002, Co 14025, Co 15015 and Co 15018 were water efficient, resulting in a lower TWF, whereas the germplasm clones *viz*. Fiji 55, ISH 111, ISH 107, Pathri, and Gungera had smaller GWF, BWF and TWF.

### Plant material availability

This study complies with local and national guidelines. Plant experiments were also performed in accordance with the relevant guidelines and regulations.

## Data Availability

The datasets and/or analysed during the current study available from the corresponding author (A. S. Tayade (arjuntayade3@gmail.com) on reasonable request. All data generated or analysed during this study are included in this published article.
