# Author Correction: Water-efficient genotypes along with conservation measures significantly reduce the green and blue water footprints in sugarcane (*Saccharum* spp.)

**DOI:** 10.1038/s41598-023-45913-7

**Published:** 2023-10-31

**Authors:** A. S. Tayade, S. Vasantha, S. Anusha, R. Arun Kumar, G. Hemaprabha, P. Geetha, V. Krishnapriya, K. Sammi Reddy, Rajan Bhatt, Manzer H. Siddiqui, Mahipal Singh Kesawat

**Affiliations:** 1 ICAR-Sugarcane Breeding Institute, Coimbatore, 641007 Tamil Nadu India; 2 ICAR-National Institute of Abiotic Stress Management, Baramati, 413115 Pune India; 3 PAU-Regional Research Station, Kapurthala, 144601 India; 4 Department of Botany and Microbiology, College of Science, King Saud University, Riyadh, 11451 Saudi Arabia; 5 Institute of Molecular Biology and Genetics, School of Biological Sciences, Seoul National University, Seoul, 08826 Republic of Korea

Correction to: *Scientific Reports* https://doi.org/10.1038/s41598-023-40223-4, published online 14 August 2023

The original version of this Article contained errors.

In the Author Contributions section,

“A.S.T. and S.V.: conceptualization, supervision, investigation, data curation, methodology. R.B., M.H.S., A.S.T. and S.V.: data curation, formal analysis, writing—original draft. M.H.S., M.S.K, G.H.: provided sugarcane commercial hybrids, species clones and resources required for experiments. M.H.S., M.S.K, K.S.R., R.B., P.G., S.A., R.A.K. and V.K.: validation, Writing—review and editing. All authors have read and agreed to the published version of the manuscript.”

now reads:

“A.S.T. and S.V.: conceptualization, supervision, investigation, data curation, methodology. R.B. and M.H.S., A.S.T. and S.V.: data curation, formal analysis, writing—original draft. G.H.: provided sugarcane commercial hybrids, species clones and resources required for experiments. M.H.S., M.S.K, K.S.R., R.B., P.G., S.A., R.A.K. and V.K.: validation, Writing—review and editing. All authors have read and agreed to the published version of the manuscript.”

In addition, the Funding section was incomplete.

“ICAR-Sugarcane Breeding Institute, Coimbatore is a Govt. Institute working under the umbrella of the Indian Council of Agricultural Research, New Delhi. ICAR-SBI is involved in the sugarcane germplasm collections, maintenance, screening for drought/salinity/disease tolerance and its further utilization in breeding programme. The research work has been taken up at the Institute as a regular activity under the mandate of Institute. No extra funding from other sources has been received for conducting the presented work. This study was supported by the Researchers Supporting Project number (RSP2023R347), King Saud University, Riyadh, Saudi Arabia.”

now reads:

The research work has been taken up at the Institute as a regular activity under the mandate of Institute. No extra funding from other sources has been received for conducting the presented work. This study was supported by the Researchers Supporting Project number (RSP2023R347), King Saud University, Riyadh, Saudi Arabia for extending funding support for this publication. The funder had no role in study design, data collection and analysis, decision to publish, or preparation of the manuscript.”

The original Article has been corrected.

